# Outbreak of digital extensor dysfunction compatible with acquired equine polyneuropathy observed for the first time in Iceland

**DOI:** 10.1186/s13028-025-00835-4

**Published:** 2025-11-26

**Authors:** Sigríður Björnsdóttir, Ólöf Guðrún Sigurðardóttir, Charlotta Oddsdóttir, Ingunn Reynisdóttir, Siv Hanche-Olsen, Gittan Gröndahl

**Affiliations:** 1 Icelandic Food and Veterinary Authority, Austurvegur 64, 800 Selfoss, Iceland; 2 Division of Bacteriology and Pathology, Institute for Experimental Pathology at Keldur, Keldnavegi 3, 112 Reykjavík, Iceland; 3 Syðri Völlum, 531 Hvammstanga, Iceland; 4 Department of Companion Animal Clinical Sciences, Faculty of Veterinary Medicine, Norwegian University of Life Sciences, Ås, Norway; 5 Department of Animal Health and Antimicrobial Strategies, Swedish Veterinary Agency, Uppsala, Sweden

**Keywords:** Demyelination, Icelandic horses, Knuckling, Locomotor symptoms, Neuropathy, Pelvic limb, Peripheral nerves, Wrapped forage

## Abstract

**Background:**

Acquired equine polyneuropathy is a neuromuscular syndrome characterized by digital extensor dysfunction, primarily affecting the pelvic limbs, with consistent, repeated knuckling. Despite being recognized as an emerging disease in Scandinavia since 1995, the aetiology remains unknown, and cases have been limited to Norway, Sweden, and Finland.

**Case presentation:**

On a combined breeding and training farm in Iceland, 30 out of 145 horses (21%) presented with acute pelvic weakness, pelvic limb digital extensor dysfunction, knuckling and/or recumbency, from May to August 2019. The affected horses, aged 2–9 years, were from four out of six free-ranging groups on the farm. All affected horses had been fed a specific batch of wrapped forage for 11 days or more, while none of the 40 stabled horses fed a different wrapped forage were affected. Eleven case horses were euthanised due to severe pelvic limb weakness, and/or recumbency, yielding a case fatality rate of 37%. Histopathological examination of peripheral nerves from one case revealed severe demyelination.

**Conclusions:**

This case report documents the first recognized outbreak of equine polyneuropathy in Iceland. Describing one of the largest documented outbreaks of the disease, this report provides crucial insights into the epidemiology and clinical manifestation in mainly untamed horses kept and fed outdoors.

**Supplementary Information:**

The online version contains supplementary material available at 10.1186/s13028-025-00835-4.

## Background

Acquired equine polyneuropathy (AEP) is a neuromuscular syndrome characterized by digital extensor dysfunction primarily of the pelvic limbs with consistent, repeated knuckling (an abnormal position where the dorsal side of one or both hooves or fetlocks touches the ground) [1–3]. The clinical manifestations of AEP vary in severity and the disease course can be either acute and fatal due to recumbency or more prolonged. Knuckling horses may remain in the abnormal limb position for less than seconds to several minutes, or they may become unable to rise from recumbency, even with assistance. Surviving horses most often return to normal function again after a convalescence period of 5–8 months [2, 4, 6]. The syndrome has emerged in Norway, Sweden and Finland since 1995, exhibiting spatio-temporal clustering of case farms [3] and correlation to feeding with certain batches of wrapped forage [1–5]. However, AEP has not been described outside these countries. The histopathological changes in AEP are characterised by de- and remyelinating large fibre polyneuropathy with hypertrophy of Schwann cell perikaria, often associated with conspicuous perinuclear inclusions [5, 6]. An unknown, feed related toxin has been suggested to be the most likely trigger for this disease, either directly or indirectly via an immuno-mediated mechanism [5, 6].

In this case report we describe an outbreak in 2019 of a clinical disease compatible with AEP, on an Icelandic farm. This disease has not previously been diagnosed in Iceland.

## Case presentation

### Case definition

The case definition for polyneuropathy in this report is a horse exhibiting sudden weakness and repeated bilateral digital extensor dysfunction of pelvic limbs, characterized by (a) a tendency to knuckle, and/or (b) obvious knuckling, and/or (c) acute paraplegia with recumbency, and (d) no signs of cranial nerve dysfunction. Cases had to have been in a common residence with other cases, and in this outbreak all cases were identified within 3 months. Restless movements of the pelvic limbs, resembling itching and proprioceptive deficits of the distal limbs, were observed in some case horses and horses without obvious pelvic limb weakness. While discussed as part of the clinical presentation of this outbreak, it was not used as an inclusion criterion. An additional horse found dead on the premises during the same period did not fulfil the inclusion criteria.

### Study population and management

All cases originated from a combined breeding and training farm in Northwest Iceland in 2019. The farm kept a total of approximately 145 horses, distributed across seven different groups, according to their nutritional requirements, age and purpose or use for riding (Table 1). Groups kept outdoors were on semi-natural pastures with varying amounts of withered grown grass from previous summer [7], except for one group kept on soil in a paddock. Some of the horses in training were transported to and from another stable in the capital area in Southwest Iceland, with shared ownership.

**Table 1:** Overview of groups involved in the first Icelandic outbreak of acquired equine polyneuropathy in 2019

Group	Group definition	Number of horses May 6th	Age range	Exposure to forage A^1^	Access to other feed	Cases (n)	Attack rate (%)	Dead (n)	Case fatality rate (%)
1	Broodmares	20	6–20y	4w	Winter pasture^2^	3	15%	0	0%
2	Youngsters (mares and geldings)	21	2–3y	6w	Limited^3^	14	66.7%	6	43%
3	Yearlings	19	1y	4w^4^	Limited	0	0	0	0
4	Stallions	10^5^	2–7y	4w	Limited	8	80%	4	50%
5	Transient (mares and geldings)	7^6^	4–8y	4w	None	5	71%	1	20%
6	Other horses (big field)	Approx. 28	4–25y	6w^7^	Winter pasture^2^	0	0%	0	0
7	Stabled horses	40	4-ND y	0w	Forage B and mineral supplement^8^	0	0%	0	0
	Total	Approx 145				30	21%	11	37%

^1﻿^Forage A was used during 1 April to 14 May 2019

^2﻿^Pasture with withered grass from the previous summer

^3﻿^Pasture with short, withered grass from previous summer

^4﻿^Yearlings separated from their mothers in February, stabled and fed Forage B until 15 April when feeding with forage A commenced. Male yearlings were then moved to a field with limited withered grass on 7 May 2019 and feeding with Forage A ceased on 9 May

^5﻿^One stallion out of ten had been stabled from 11 April 2019 but previously fed Forage A for eleven days

^6﻿^Two horses out of seven were transported to a stable in Southwest of Iceland 14 May 2019

^7﻿^Restricted feeding of Forage A compared to other groups

^8﻿^Kalksalt. Kalksalt ehf. www.hafnarbakki, 425 Flateyri, IS. kalksalt.is

Horses affected by acquired equine polyneuropathy in the first described outbreak of the disease in Iceland. The table shows group definition, number of horses in each group, age range, length of exposure to a certain batch of wrapped forage (Forage A) and other feed, numbers of cases, as well as ratio of cases and case fatalities in each group. The first case was noticed on 6 May 2019, in group 2. Groups 1–6 were fed outdoors with varying access to previous year’s withered grass, whereas group 7 consisted of stabled horses that were fed only Forage B. Group 5 was of changing composition, as horses were moved to and from the group during the feeding of Forage A. y: years old; w: weeks; ND: no data

The broodmares, and another group of adult horses not in use for riding, groups 1 and 6 (Table 1), were kept on big fields of winter pasture, while the young horses in groups 2–4 were kept on smaller fields with limited winter pasture. Group 5 was kept outside in a paddock on soil, without access to grazing. In the first week of May 2019, there was hardly any fresh grass available due to a cold spring. All horses kept outside at that time (groups 1–6) were fed the same crop of wrapped forage sourced from a newly cultured field (first year of use), cut on 20 July 2018, referred to as Forage A. The feeding quantity was inversely proportional to access to grazing. In contrast, horses in training were stabled indoors at the farm (group 7 in Table 1) and fed another wrapped forage sourced from an adjacent field, also newly cultured but in the second year of use and cut on 30 June 2018, referred to as Forage B. Yearlings (group 3) had been separated from their mothers in February and stabled for 2 months and fed with Forage B. On 15 April they were moved to a field with limited winter pasture and transitioned to Forage A. The male yearlings were separated from the females on 7 May and moved to a field with new grass, where feeding with Forage A was discontinued after two days.

### Case history

In total, 30 horses (21% of total) met the defined case criteria. Findings are described in detail below and in Table 2. Each case horse was observed with one or more of the various degrees of knuckling previously described in polyneuropathy [2], from tendency to knuckle to knuckling for shorter or longer time or recumbency. Two of the case horses were found recumbent without prior clinical signs, but in spatio-temporal proximity to other cases. The case fatality was 11 out of 30 horses (37%), all euthanized due to prolonged recumbency or other welfare concerns (Tables 1 and 2). The 30 cases were identified during a 3-month period from May to August 2019, with 88% of the cases appearing within three consecutive weeks in May (Table 2).

**Table 2:** Description of 30 cases in the first outbreak of acquired equine polyneuropathy in Iceland 2019

Case no	Age	Gender	Date of signs noticed	Group	Clinical signs	Outcome
1	2y	Mare	6 May	2	Pelvic limb weakness. Tendency to knuckling (retrospectively on video). Recumbency	Euthanized (8 May)
2	3y	Gelding	14 May	2	Pelvic limb weakness. Knuckling. Recumbency	Euthanized (15 May), PM*
3	2y	Gelding	14 May	2	Pelvic limb weakness. Repeated knuckling	Euthanized (20 May), PM**
4	2y	Mare	14 May	2	Pelvic limb weakness. Knuckling. Recumbency	Euthanized (14 June)
5	2y	Mare	14 May	2	Pelvic limb weakness. Knuckling. Recumbency	Euthanized (14 June)
6	6y	Mare	16 May	5	Recumbency	Euthanized (16 May)
7	7y	Gelding	16 May	5^1^	Falling when ridden. Pelvic limb weakness. Knuckling	Recovered
8	6y	Gelding	16 May	5^1^	Pelvic limb weakness. Knuckling	Recovered
9	3y	Gelding	16 May	2	Pelvic limb weakness. Knuckling. Recumbency	Euthanized (14 June)
10	3y	Mare	16 May	2	Pelvic limb weakness. Tendency to knuckling	Recovered
11	3y	Gelding	16 May	2	Pelvic limb weakness. Tendency to knuckling	Recovered
12	2y	Mare	16 May	2	Recumbency	Recovered
13	3y	Gelding	16 May	2	Pelvic limb weakness. Tendency to knuckling	Recovered
14	2y	Mare	16 May	2	Pelvic limb weakness. Repeated knuckling	Recovered
15	2y	Gelding	16 May	2	Pelvic limb weakness. Tendency to knuckling	Recovered
16	2y	Mare	17 May	2	Pelvic limb weakness. Tendency to knuckling	Recovered
17	2y	Gelding	17 May	2	Pelvic limb weakness. Tendency to knuckling	Recovered
18	7y	Mare	17 May	5^2^	Pelvic limb weakness. Repeated knuckling	Recovered
19	4y	Stallion	17 May	4	Pelvic limb weakness. Repeated knuckling. Muscle atrophy. Recumbency	Euthanized (22 May)
20	7y	Stallion	18 May	4	Pelvic limb weakness. Repeated knuckling. Muscle atrophy. Recumbency	Euthanized (22 May)
21	3y	Stallion	18 May	4	Pelvic limb weakness. Repeated knuckling. Muscle atrophy. Recumbency	Euthanized (22 May)
22	2y	Stallion	18 May	4	Pelvic limb weakness. Tendency to knuckling	Recovered
23	4y	Stallion	18 May	4	Pelvic limb weakness. Tendency to knuckling	Recovered
24	4y	Stallion	27 May	4^3^	Pelvic limb weakness. Tendency to knuckling	Recovered
25	5y	Mare	14 June	1	Pelvic limb weakness. Tendency to knuckling	Recovered
26	7y	Mare	14 June	1	Pelvic limb weakness. Tendency to knuckling	Recovered
27	7y	Mare	14 June	1	Pelvic limb weakness. Tendency to knuckling	Recovered
28	4y	Stallion	27 June	4^4^	Knuckling. Unable to walk	Euthanized 28 June
29	9y	Mare	15 July	5	Tendency to knuckling	Recovered
30	3y	Stallion	1 August	4	Tendency to knuckling	Recovered

^1﻿^Stabled in Southwest Iceland when clinical signs were noticed

^2﻿^Moved to group 6, 14 May

^3﻿^Had been stabled and fed Forage B from 11 April

^4﻿^Had been brought to another farm for breeding the previous day

^*^Full post-mortem examination

^**^Partial post-mortem examination, pelvic limbs

Thirty recorded cases of acquired equine polyneuropathy on a horse farm in Iceland in May–August 2019. The table describes each case, including age, gender and group in which each horse was kept and fed, date for first clinical signs, nature of clinical signs and outcome. The total number of horses were approximately 145, and grouped as follows: Group 1, broodmares; group 2, youngsters (2–4y); group 3, yearlings (1y); group 4, stallions; group 5, transient (horses being moved to and from the group); group 6, other horses kept on pasture; group 7, stabled horses

**Fig. 1:**
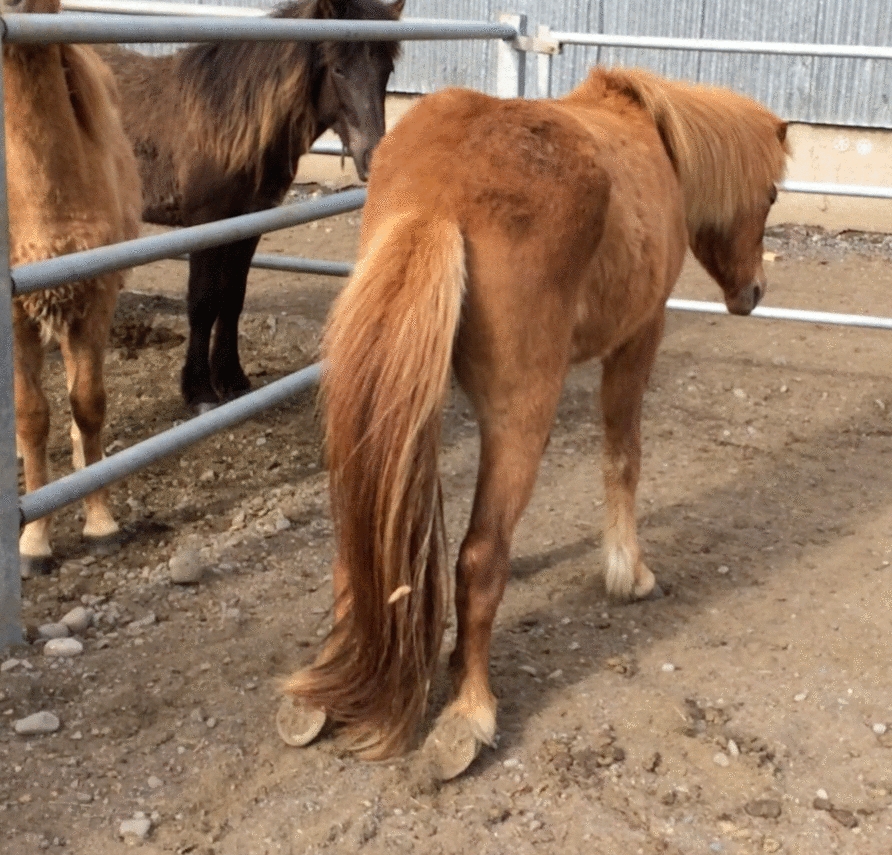
Two-year-old gelding (case 3) presenting bilateral knuckling

The first case was identified on 6 May 2019, when a 2-year-old mare in group 2 was found standing in the field with a stiff back, alternately lifting the pelvic limbs (case 1). Laboured breathing (dyspnoea) was noticed, and the mare expressed discomfort through constant tail-sweeping. After walking/running from the field to the stable, some 400–500 m the subsequent day, severe pelvic limb weakness and mobility issues became apparent. The mare repeatedly attempted to lie down. She showed full consciousness, normal appetite, and normal rectal temperature. By the next evening, she was unable to rise and was euthanized for humane reasons on 8 May. Although not noticed at the time, knuckling was recognized retrospectively through video analysis. When four additional horses in group 2 were identified with weakness in the pelvic limbs, exhibiting varying degrees of paresis/dysfunction of metatarsophalangeal extensor muscles and knuckling of the metatarsophalangeal joints on 14 May (cases 2–5), botulism was initially suspected. Listeriosis and myeloencephalopathy due to EHV-1 were regarded as differential diagnoses, although unlikely as the latter has never been diagnosed in Iceland. Consequently, the feeding regimen was immediately changed from Forage A to Forage B for all horses at the farm. Other horses in group 2 were moved to a field closer to the stable the same day. Case 2 was euthanized 15 May due to recumbency and subjected to postmortem examination. During the subsequent days, although the affected horses appeared weak and displayed neurological signs, the initial suspicion of botulism was dispelled due to the absence of cranial nerve deficits and other typical clinical signs associated with equine botulism, such as decreased muscle tone of the tongue, eyelid or tail and sudden death. Therefore, AEP became the tentative diagnosis, as repeated bilateral knuckling of pelvic limbs was observed in several horses. Case 3 was euthanised 20 May due to repeated knuckling and poor prognosis (Fig. 1). The limbs, cut just above the carpus and tarsus, were sent for postmortem examination at the Institute for Experimental Pathology at Keldur.

Eighteen cases (cases 6 to 23) from groups 2, 4 and 5 were identified within three days, from 16 to 18 May (Table 2). On 16 May, a 6-year-old mare from group 5 was discovered in recumbency and stuck in a small ditch (case 6). After rescue, the mare was unable to rise and was consequently euthanized. On the same day, a 7-year-old horse from group 5 (case 7), stumbled and fell while being ridden. This horse had been transported to the stable in the Southwest two days earlier. Weakness in the hind quarters was identified, especially noticeable when walking the horse in and out of the stable box. Another horse that had been transported to the same stable from the same group (case 8, 6-year-old) displayed similar clinical signs. Both horses were transported back to the farm a few days later where they were added to group 6 for recovery. Further observation revealed knuckling in both cases 7 and 8. No other horses in the stable in Southwest Iceland, where they had visited, became affected.

Cases 9–17 involved 2- and 3-year-olds from group 2, identified on 16 and 17 May, exhibiting weakness in the pelvic limbs with varying degrees of knuckling, and recumbency. Eight of the horses recovered, but one remained recumbent and was euthanized a month later (case 9).

On 14 May, two mares from group 5 were moved to a larger field with the horses in group 6. On 17 May, one of these mares, a 7-year-old, displayed severe clinical signs (case 18). The other mare, a 6-year-old, went missing in heavy fog that day and was found dead the following day. Due to uncertainty of the clinical signs before she died, she was not included as a case.

The first stallion in group 4 exhibited clinical signs on 17 May (case 19) and was consequently brought from pasture by trailer. When another stallion was suspected the day after (case 20), the entire group 4 was brought to the stable to reduce their level of activity. During the move, three additional stallions were observed with abnormal gait consistent with AEP (cases 21–23). Despite having a normal appetite, the body condition score of three stallions (cases 19, 20, and 21) dropped from 3 to 2 within two weeks, on a scale of 1–5 where a score of 3 represents normal riding condition [8]. These horses had noticeable loss of muscle mass, particularly in the gluteus muscles, and became recumbent which led to euthanasia on 22 May. Nine days later another case in group 4 (case 24) was noticed. This stallion had been stabled since 11 April and therefore only fed Forage A for eleven days.

On 14 June, as most of the broodmares in group 1 had foaled, they were moved a 2 km distance to the stable area. During the walk/run home, knuckling was noticed in three of the youngest mares (cases 25–27), whereas no signs were detected in the rest of the group.

In the middle of June, many horses were observed repeatedly and unusually scratching their pelvic limbs. Some of them were also keeping one of the pelvic limbs lifted, alternately, indicating proprioceptive deficits. An additional movie file shows this in more detail [see Additional file 1]. These included most of the case horses with severe signs of digital extensor dysfunction, as well as female yearlings that never developed clinical signs of weakness or knuckling.

At the end of June, a 4-year-old stallion in group 4, initially displaying signs of itching in the limbs but no signs of weakness, was brought to another farm for breeding. The stallion was found knuckling and unable to walk the following day, and he was euthanized the day after (case 28). The last cases were identified when horse training had resumed. Repeated knuckling was observed in a 9-year-old mare in mid-July (case 29) and two weeks later in a 3-year-old stallion (case 30), groups 5 and 4, respectively.

At the end of August, the situation was considered stable, with no new cases or worsening of clinical signs in the survivors. By December 2019, considerable improvement in clinical signs was observed for all 19 survivors, and full recovery was achieved within a year after onset of clinical signs. Follow up in 2024, 5 years later, revealed that 17 survivors were in use for riding or breeding while two had been slaughtered for reasons unrelated to the disease.

### Case fatalities and postmortem examinations

Among the 30 cases, a total of 11 case horses were euthanized due to their disease, after a course ranging from 0–6 days (8 horses, cases 1, 2, 3, 6, 19, 20, 21 and 28), to 1 month (3 horses, cases 4, 5, 9) (Table 2).

The first four horses were euthanized between 8 and 20 May (cases 1–3 and 6). Two of them exhibited knuckling followed by recumbency, one was found recumbent without previous signs and one horse was euthanized due to persisting, severe knuckling (Fig. 1, Table 2). Later in May, three stallions in group 4 (cases 19–21) were euthanized as they were unable to rise for more than 24 h. Further three horses from group 2 (cases 4, 5 and 9) were euthanized between 12 and 14 June, and a stallion from group 4 on 28 June (case 28).

Postmortem examination was performed on the carcass of one horse and the limbs of another (cases 2 and 3 respectively). Autopsy of case 2 did not reveal gross pathological changes, and histopathological examination of the brain and spinal cord ruled out listeriosis and myeloencephalopathy due to EHV-1 infection. PCR for EHV-1 (brain tissue and nasal swab, case 2) was also negative. At the time of examination, suspicion of peripheral neuropathy had not arisen and hence no peripheral nerves were examined.

**Fig. 2:**
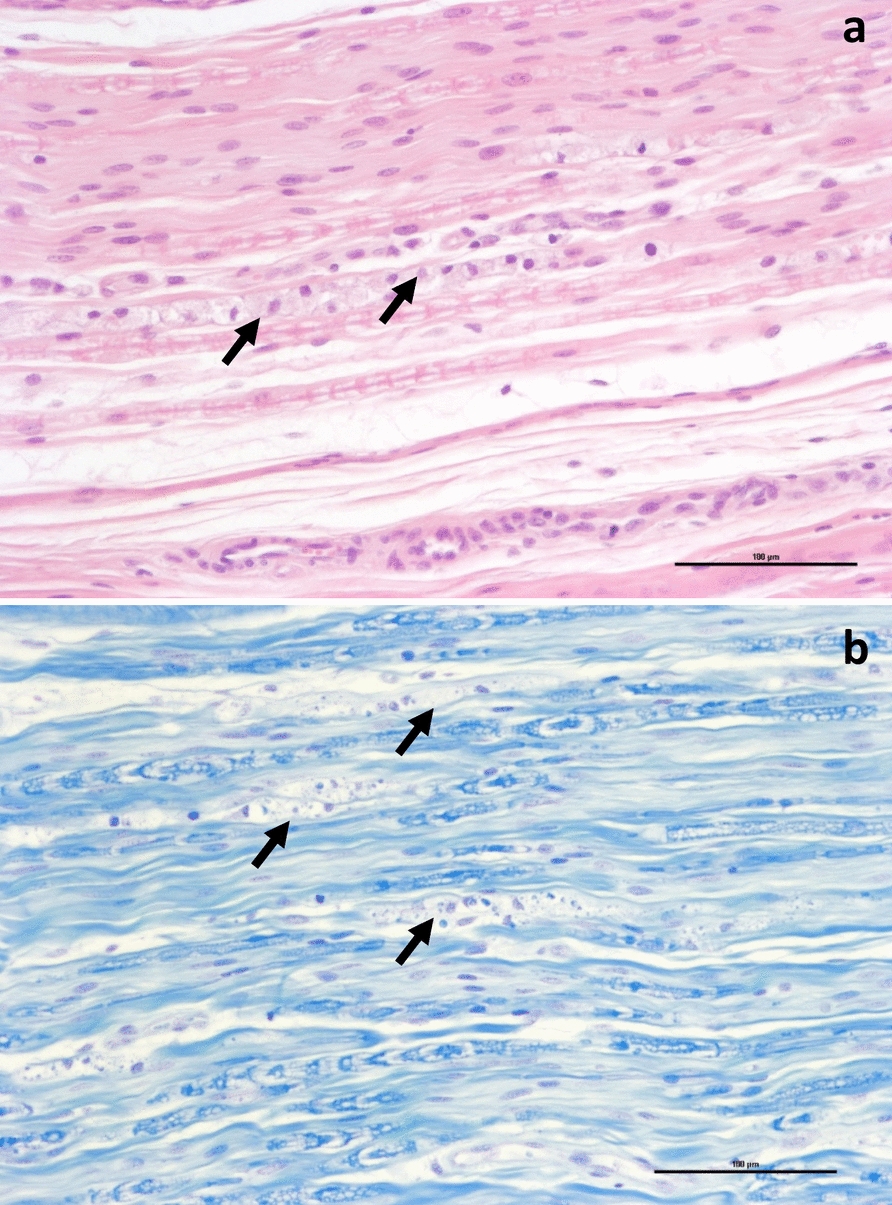
Histological sections of peripheral nerves from the pelvic limb. Sections were taken from the lateral digital nerve on the left pelvic limb of the 2-year-old gelding constituting case 3. **a** Left digital nerve showing chains of digestion chambers with foamy macrophages (arrows), and cords of proliferating spindle-shaped cells. HE. Bar = 100 μm. **b** Same nerve as in figure a, with fragmentation and loss of myelin (arrows). Luxol fast blue. Bar = 100 μm

**Fig. 3:**
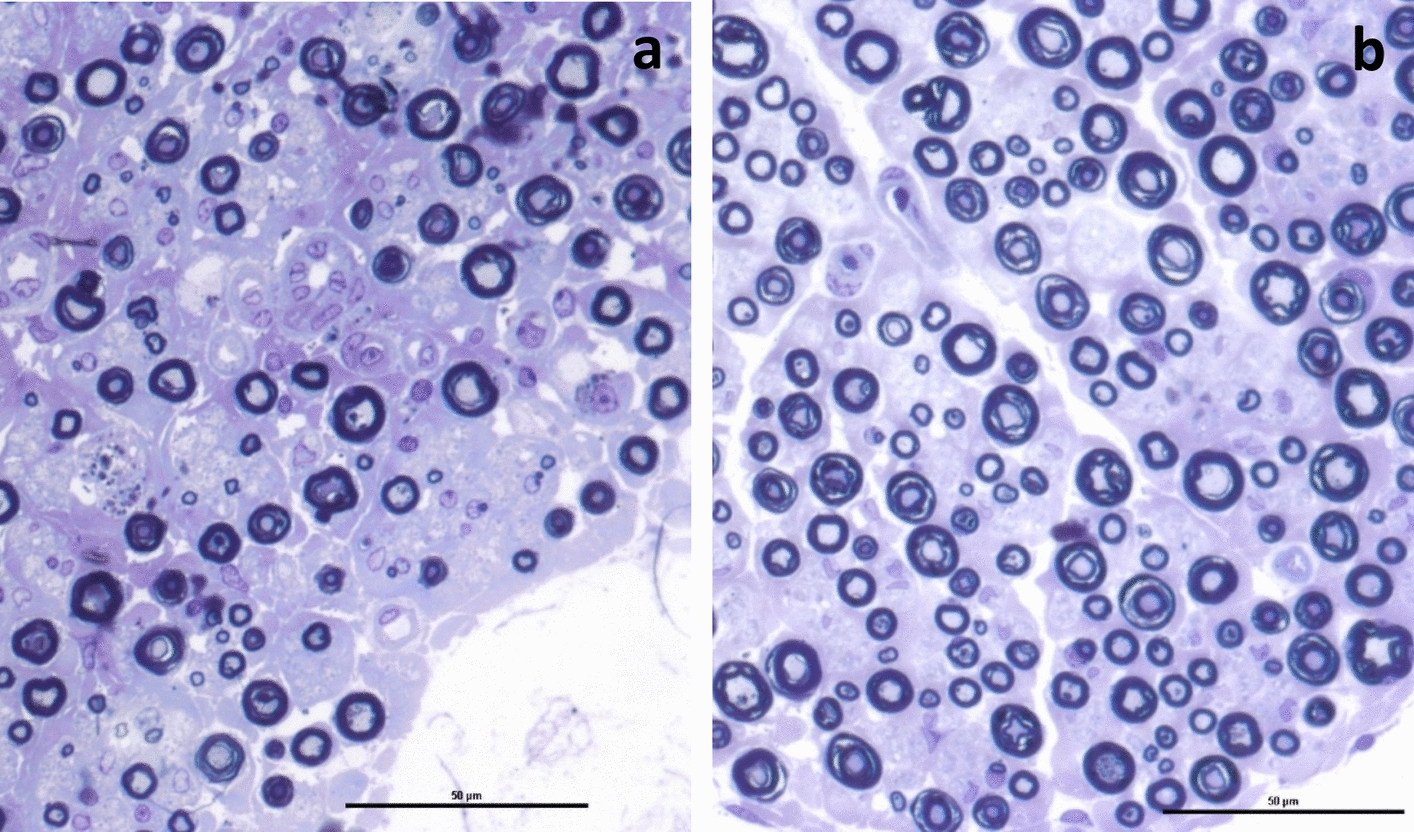
Histological sections of peripheral nerves from equine pelvic limbs. Sections were taken from the lateral digital nerve on the left pelvic limb of **a** the 2-year-old gelding constituting case 3 and **b** a 6-year-old healthy horse from a different herd, which was euthanized for other reasons. **a** Nerve fascicle with severe loss of myelinated nerve fibres and fragmented myelin. Semithin section, toluidine blue. Bar = 10 µm. **b** Normal nerve fascicle showing nerve fibres of varying width, with varying number of myelin layers around them

Peripheral nerves were dissected from the limbs of the horse in case 3, including median and lateral digital nerves of the thoracic limbs, and the fibular, tibial and lateral digital nerves of the pelvic limbs. The samples were formalin-fixed, routinely processed, and paraffin-embedded. Sections were stained with haematoxylin–eosin (HE) and luxol fast blue (LFB). In addition, semithin resin sections of the lateral digital nerves of both pelvic limbs were stained with toluidine blue. All nerves examined histologically exhibited lesions to varying degrees, with more severe lesions in the nerves of the pelvic limbs. Axons were unevenly and poorly myelinated, with multifocal vacuoles in the myelin sheaths containing foamy macrophages and fragmented myelin. Interstitially, an increased amount of spindle-shaped mesenchymal cells was observed (Fig. 2a) and fragmentation and loss of myelin (Figs. 2b and 3a). There was also a mild, multifocal inflammation in the epineurium with a mixture of lymphocytes, plasma cells and granulocytes, including occasional eosinophilic leukocytes.

As part of a study on anti-ganglioside immunoglobulins involvement in AEP horses, sera from 8 horses were analysed. All 8 were tested for IgM antibodies and were seropositive to gangliosides GM1 (6/8), GM2 (8/8), GD1a (7/8), GD1b (8/8) and GQ1b (7/8) but not to Myelin-Associated Glycoprotein, MAG (personal communication, G. Gröndahl).

## Discussion and conclusions

This first outbreak in Iceland of digital extensor dysfunction associated with degenerative lesions in myelin sheaths and axons of peripheral nerves in horses shares the key clinical and epidemiological characteristics previously described for AEP in Norway, Sweden and Finland. These common features include bilateral knuckling of metatarsophalangeal joints, pelvic limb weakness, abnormal gait and/or recumbency, as well as spatial and temporal clustering. Additionally, the outbreak occurred in the month of May, coinciding with the described seasonal pattern for AEP [3]. Furthermore, there was an association of cases with a certain batch of wrapped forage as observed in AEP [1–3]. The affected individuals were relatively young, with 29 out of 30 falling within the age range of 2–7 years, of which 22 were between 2 and 4 years old.

This aligns with a risk factor study for AEP, indicating that horses younger than 12 years are at higher risk [2]. The prevalence on farm level was 21% and the case fatality rate was 37%, corresponding to findings from a study involving 13 farms with AEP in Norway and Sweden [2]. In that study, the prevalence ranged from 11 to 71% at the farm level (mean 24%) and the mean case fatality rate was 29% (0–100%) [2]. Higher prevalence (65%) and case fatality rate (64%) were reported in an outbreak in Finland [6]. All Icelandic cases manifested within 87 days, a timeline consistent with reports of AEP in Scandinavia, where the median time was 56 days (3–100; n = 11 farms) [2].

In this outbreak, all case horses were kept outdoors and fed a single type of forage, whereas no cases were observed among stabled horses fed a different forage. Previous studies have shown that horses in various indoor/outdoor housing combinations can be affected by AEP. However, multivariable analyses from multiple affected farms, where all horses on each farm were fed the same forage, suggest that loose housing outdoors may be more commonly associated with the condition compared to box stabling [2].

Polyneuropathy was confirmed by histopathology in one clinical case, whereas other initial differential diagnoses such as EHV-1, listeriosis, and equine botulism did not correspond with the clinical course or microbiological and pathological findings. In addition, recent studies have shown a significantly higher levels of serum IgM antibodies to tested antigangliosides in horses involved in AEP outbreaks, as seen in 8 of the tested horses in this outbreak. These results point to an immune-mediated component in the pathogenesis and correspond to findings in Norwegian and Swedish horses with AEP [9].

More advanced neurological diagnostic methods, such as transcranial magnetic stimulation to assess increased motor potential onset latency times has recently been described in horses with clinical and subclinical AEP [10], however, these methods were not available in this study.

The presence of the most critical clinical signs, consistent knuckling, may have been underestimated as the study is primarily based on observations of untrained horses in free locomotion. Unnecessary interventions were avoided to mitigate the risk of stress, which could have adversely impacted their prognosis. This limited the use of traditional neurological examinations and provocation tests. However, some horses displayed knuckling after a trailer-ride or running for a distance between pastures/stable, in accordance with observations in AEP cases.

In this outbreak, one horse was found dead, similar to an outbreak of AEP reported by Gröndahl et al. [2]. In that report, the horse died during the night following the first day of observed clinical signs of weakness. In the current case, a pasture mate had shown signs the same day, but the deceased horse had not, making it a plausible but unconfirmed case. A possible explanation for death in paretic horses is that recumbency, struggling and awkward body positions can lead to distress with rapid deterioration and a fatal outcome [11].

In addition to clinical signs of equine polyneuropathy previously described in Scandinavia, this outbreak notably featured frequent pruritus of the distal pelvic limbs, without any signs of parasites or dermatitis. The itching or pruritus served as a precursor to or concomitant to digital extensor dysfunction in case horses, while in others, not fulfilling case criteria, the abnormal itching behaviour manifested without apparent locomotor dysfunction. This was especially prominent among yearling fillies in group 3. Neuropathic itch provides a reasonable explanation, since it includes dysesthesias like stinging, tingling, or sensations resembling electroshocks [12]. Lesions in any site of the somatosensory system, including peripheral nerve fibres, nerve plexuses and ganglia, and centrally the spinal cord, brainstem, thalamus or cortex, may lead to neuropathic itch [13]. Itching has been reported in approximately 30% of human patients with peripheral neuropathies [14].

In accordance with the earlier presented but not proven theory that forage plays a role in AEP [2], all cases in this outbreak of polyneuropathy were associated with a certain batch of wrapped forage, Forage A. All the cases were among horses that were exposed to Forage A, while none of the 40 horses that were only fed Forage B and stabled the whole season became affected (group 7; Table 1). The attack rate of clinical signs within a group varied from 0 to 71.4% in groups that received Forage A. The highest attack rates (66.7–71.4%) and all fatalities (case fatality rates 20–50%) were observed in three groups that had been fed Forage A for 4–6 weeks (group 2, 4, 5; Table 1), with the exception of case 24 that had access to Forage A only for 11 days before being stabled and showed knuckling 2 months later. These three groups had limited or no access to winter pasture. A less affected group included the broodmares, which were fed Forage A for 4 weeks concurrently with access to winter pasture. They exhibited an attack rate of polyneuropathy of 15% and no fatalities (group 1, Table 1). An attempt to analysis of the suspected feed related toxin was not performed due to lack of target and unfortunately, the leftover of forage A was destroyed.

Clinical signs were detected while the suspected feed was still in use or within a week after changing forage in most cases (23/30). Seven cases that first displayed neurological signs 2–10 weeks after the change to forage B mostly showed milder signs, with one exception. In two groups of horses (groups 3 and 6), no knuckling was observed despite exposure to Forage A for 4–6 weeks. Adult horses in group 6 had been kept mainly on winter pasture with more restricted feeding (Table 1), therefore consuming less of Forage A. On the other hand, the yearlings in group 3 were reliant on the putative risk factor Forage A for four weeks, as they only had access to limited winter pasture. It is difficult to draw a definitive conclusion regarding this finding, but a few speculative factors may contribute, including that a putative feed-related trigger probably would cause dose-dependent effects or might be unevenly distributed in a crop or bale. In addition to the variation of the exposure to the suspected forage, other possible protective or risk factors may have affected the variation in clinical signs in this outbreak. Intrinsic factors such as age and gender were confounding with the exposure and could not be analysed separately.

The prognosis for affected horses is highly dependent on the management (other sources of food) and probably also the possibilities of intensive care in a holding. This study revealed survival rate of 63% where all survivors received full recovery.

Wrapped forage has been used to feed the native Icelandic horse population (around 70,000 horses) outside of the grazing season for the past 30 years, with no reported outbreaks of polyneuropathy with knuckling. Given the variation in the clinical manifestation demonstrated in this study, it is, however, possible that outbreaks might have been overlooked in Iceland in the past, particularly if there were only few affected horses, mild clinical signs and/or less dramatic outcomes.

In conclusion, this case report highlights one of the largest documented outbreaks of acquired polyneuropathy in horses, compatible with AEP in Scandinavia. The report provides crucial insights into the epidemiology and clinical manifestation in mainly untamed horses kept and fed outdoors.

## Supplementary Information

Additional file 1

## Data Availability

The datasets used during the current study are available from the corresponding author on reasonable request.
